# The Pathology of the Contracted Granular Kidney

**Published:** 1897-03

**Authors:** 


**The Pathology of the Contracted Granular Kidney.** By Sir George Johnson, M.D., F.R.S. Pp. viii., 62. London: J. & A. Churchill. 1896.

This little volume gives an excellent outline of the two most characteristic points in the teaching of the master who has recently been removed from us. The stop-cock action of the muscular-walled arterioles was much emphasised by Johnson more than thirty years ago, and is now accepted as a factor in the production of hypertrophy of the left ventricle of the heart, as well as in explanation of the phenomena of Raynaud's disease: the muscular action of the pulmonary arterioles in cases of apnœa and in the collapse stage of cholera were favourite topics with Johnson for many years.

The connection between the arterial changes, the cardiac hypertrophy, and the granular kidney is now universally admitted; and that the wasting of the granular kidney is the result of pathological changes which are primarily and essentially *intra-tubular* was always stoutly maintained by the author, who insisted upon the view that the pathological process is neither primarily interstitial nor of the nature of inflammation.

Few investigators have been left so completely masters of the field as was Sir George Johnson in his spirited controversies on the nature of the various pathological conditions of the kidney.

